# Prevalence of hepatitis E infection in HIV/HCV-coinfected patients in Spain (2012–2014)

**DOI:** 10.1038/s41598-018-37328-6

**Published:** 2019-02-04

**Authors:** Sonia Vázquez-Morón, Juan Berenguer, Juan González-García, Ma Ángeles Jiménez-Sousa, Isabel Canorea, Josep M. Guardiola, Manuel Crespo, Carmen Quereda, José Sanz, Ana Carrero, Victor Hontañón, Ana Avellón, Salvador Resino

**Affiliations:** 1 Laboratorio de Referencia e Investigación en Hepatitis Víricas, Centro Nacional de Microbiología, Instituto de Salud Carlos III, Majadahonda, Madrid, Spain; 2 Unidad de Enfermedades Infecciosas/VIH, Hospital General Universitario “Gregorio Marañón”, Madrid, Spain; 3 Instituto de Investigación Sanitaria del Gregorio Marañón, Madrid, Spain; 4 Unidad de VIH, Servicio de Medicina Interna, Hospital Universitario “La Paz”/IdiPAZ, Madrid, Spain; 5 Hospital Santa Creu i Sant Pau, Barcelona, Spain; 6 Complexo Hospitalario Universitario, Fundación IIS Galicia Sur, Vigo, Pontevedra, Spain; 7 Hospital Universitario Ramón y Cajal, Madrid, Spain; 8 Hospital Universitario Príncipe de Asturias. Alcalá de Henares, Madrid, Spain

## Abstract

Hepatitis E virus (HEV) has emerged as a relevant pathogen for HIV-infected patients. However, there is scarce data on HEV infection in HIV/HCV-coinfected individuals with advanced fibrosis, which seems to increase the risk of HEV infection and worsen the prognosis of liver disease. We aimed to determine the prevalence of anti-HEV antibodies, acute hepatitis E, resolved hepatitis E, and exposure to HEV in HIV/HCV-coinfected patients and to evaluate associations with clinical and epidemiological characteristics. We performed a cross-sectional study on 198 HIV/HCV-coinfected patients, 30 healthy controls and 36 HIV-monoinfected patients. We found a low concordance between techniques used for detection of anti-HEV antibodies (ELISA versus Immunoblot), particularly in HIV/HCV-coinfected patients. HIV/HCV-coinfected patients showed the highest prevalence of IgG against HEV, resolved hepatitis E, and exposure to HEV (19.2%, 17.2%, and 22.2% respectively). However, we did not find any samples positive for HEV-RNA nor significant differences between groups. Moreover, HIV/HCV-coinfected patients with CD4 T-cells <350 cells/mm^3^ had higher prevalence for anti-HEV IgG antibodies, resolved hepatitis E, and exposure to HEV than healthy controls or those with CD4 T-cells ≥ 350 cells/mm^3^ (*p* = 0.034, *p* = 0.035, and *p* = 0.053; respectively). In conclusion, HIV/HCV-coinfected patients in Spain have a high prevalence for IgG anti-HEV antibodies, resolved hepatitis E, and exposure to HEV; particularly patients with CD4+T-cells <350 cells/mm^3^.

## Introduction

Hepatitis E virus (HEV) is transmitted by the fecal-oral route (contaminated water/food transmission), causes self-limiting hepatitis in humans without significant clinical implications in most cases, and is responsible for hepatitis E outbreaks worldwide^1^. HEV is the most common cause worldwide of acute viral hepatitis with an estimated prevalence of 20 million, with 3.3 million symptomatic cases and 44,000 deaths per year^2^. In Europe, HEV infection has been described as an emergent viral hepatitis in the last years^3^, with seroprevalences ranging from 0.6 to 52.2%, as showed by a recent meta-analysis. In acute HEV infection, anti-HEV IgM antibodies are detected at the time of clinical onset (3–4 days from the onset of jaundice) and remain detectable for 3–12 months. Anti-HEV IgG antibodies appear shortly after the IgM antibody response and persist for years^4^.

HEV prevalence in HIV-infected patients from European countries is from 0.95 to 26%, depending on the geographical location and study population. These rates are similar or higher than in the general population^1,2,5^, particularly for those with low CD4+ T-cell counts^6^. In this context, HEV seroprevalence ranged from 9% to 26% in Spanish HIV-infected patients^7–13^. Therefore, HEV has emerged as a relevant pathogen for HIV-infected patients, mainly ones with low CD4+ T-cell counts or immunosuppression due to solid organ or bone marrow transplant^6^. In this regard, HIV-infected patients coinfected with HEV constitute a high-risk population for developing chronic hepatitis and the rapid progression of liver disease, but it is poorly understood in HIV-infected patients^6^.

Around 2.3 million subjects worldwide are infected with both human immunodeficiency virus (HIV) and hepatitis C virus (HCV)^14^. There is little data on HEV infection in HIV/HCV-coinfected individuals with advanced fibrosis, which seems to increase the risk of HEV infection and worsen the prognosis of liver disease^1^. Therefore, HIV/HCV-coinfected individuals constitute a population of particular interest since they have chronic hepatitis C with different stages of liver disease and a deregulated immune response that increases depending on the severity of liver disease^15^.

In this study, we aimed to determine the prevalence of anti-HEV antibodies, acute hepatitis E, resolved hepatitis E, and exposure to HEV in HIV/HCV-coinfected patients and to evaluate associations with clinical and epidemiological characteristics.

## Results

### Patients

The characteristics of the HIV/HCV-coinfected patients are shown in Table 1. Overall, the median age was 49 years, 76.8% were males, 49.7% had high alcohol intake, 79.1% acquired HIV by intravenous drug use, 29.1% had prior AIDS-defining conditions, and 98% were on combination antiretroviral therapy. Furthermore, 19.4% had CD4^+^ T-cell <350 cells/mm^3^, 13.8% had values of HIV RNA >50 copies/mL, 49.5% were cirrhotic, 71.1% were HCV-GT1 and 70.9% had HCV RNA >850,000 IU/mL. Table 1 shows the characteristics of the two control groups. The healthy controls were negative for HIV, HCV, and HBV infection. The HIV-monoinfected patients without HCV and HBV infection had undetectable HIV viral load and CD4 + > 500 cells/mm^3^, and all subjects were infected with HIV bisexual transmission (14 heterosexual and 22 homosexual).

**Table 1:** Clinical and epidemiological characteristics of the subjects includes in the study.

	Healthy controls	HIV-monoinfected patients	HIV/HCV-coinfected patients
No.	30	36	198
Age (years)	50.5 (47–53)	50 (46–52)	49 (46–52)
Gender (male)	15 (50.0%)	23 (63.9%)	152 (76.8%)
BMI (kg/m^2^)	24.97 (23.0–27.5)	25.28 (23.5–26.67)	24.39 (21.85–26.93)
BMI ≥ 25 (kg/m^2^)	13 (48.1%)	19 (54.3%)	83 (43.5%)
High alcohol intake	—	1 (3.4%)	98 (49.7%)
HIV acquired by IVDU	—	0 (0%)	155 (79.1%)
Prior AIDS	—	12 (33.3%)	57 (29.1%)
Years since HIV infection	—	—	22 (18–26)
Years since HCV diagnosis	—	—	21 (17–23)
Previous HCV therapy (IFNα + rib)	—	0 (0.0%)	90 (45.7%)
Antiretroviral therapy	—	36 (100%)	192 (98.0%)
Non-treated	—	0 (0.0%)	5 (2.6%)
PI-based	—	8 (22.2%)	30 (15.4%)
2NRTI + II-based	—	4 (11.1%)	44 (22.6%)
2NRTI + PI-based	—	0 (0.0%)	39 (20.0%)
2NRTI + NNRTI-based	—	21 (58.3%)	61(31.3%)
Others	—	3 (8.30%)	16 (8.20%)
HIV markers	—		
Nadir CD4+ T-cells	—	210 (114–343)	165 (84–250)
Nadir CD4+ T-cells <200 cells/mm^3^	—	13 (39.4%)	117 (62.9%)
CD4+ T-cells	—	832 (685–1045)	527 (384–792)
CD4+ T-cells <350 cells/mm^3^	—	0 (0.0%)	38 (19.4%)
HIV-RNA >50 copies/mL	—	0 (0.0%)	27 (13.8%)
HCV markers	—	—	
HCV genotype (N = 197)	—	—	
1	—	—	140 (71.1%)
2	—	—	4 (2.0%)
3	—	—	35 (17.8%)
4	—	—	18 (9.1%)
Log_10_ HCV-RNA (IU/mL)	—	—	6.38 (5.84–6.77)
HCV-RNA >850.000 IU/mL	—	—	157 (80.9%)
Non-invasive fibrosis indexes	—	—	
FIB-4	1.08 (0.85–1.21)	1.05 (0.88–1.34)	2.42 (1.51–4.04)
FIB-4 >3.25	0 (0%)	0 (0%)	61 (32.3%)
LSM (kPa)			
F0-F1-F2-F3 (<12.5 kPa)	—	—	100 (50.5%)
F4 (12.5–25 kPa)	—	—	52 (26.3%)
F4 (25–40 kPa)	—	—	28 (14.1%)
F4 (>40 kPa)	—	—	18 (9.1%)

Statistics: Values expressed as absolute number (percentage) and median (interquartile range).

Abbreviations: HCV, hepatitis C virus; HCV-RNA, HCV plasma viral load; HIV-1, human immunodeficiency virus type 1; LSM, liver stiffness measure; HIV-RNA, HIV plasma viral load; IVDU, intravenous drug user; AIDS, acquired immune deficiency syndrome; IFNα + rib, interferon-alpha plus ribavirin; NNRTI, non-nucleoside analogue HIV reverse transcriptase inhibitor; NRTI, nucleoside analogue HIV reverse transcriptase inhibitor; PI, protease inhibitor; II, integrase inhibitor; FIB-4: noninvasive test for liver fibrosis based on AST/ALT ratio and platelet count.

### Concordance analysis between HEV ELISA and immunoblot assays

We tested the concordance between ELISA and Immunoblot techniques for the screening of anti-HEV IgM and IgG antibodies by Cohen’s kappa test (Table 2). We found a weak concordance for anti-HEV IgM antibodies (kappa coefficient between 0 and 0.342) and moderate-good for anti-HEV IgG antibodies (kappa coefficient between 0.526 and 0.718). HIV-monoinfected patients and HIV/HCV-coinfected patients had the highest kappa coefficient values.

**Table 2:** Summary of concordances between ELISA and immunoblot assays for IgM and IgG against HEV in the two control groups (healthy controls and HIV-monoinfected patients) and HIV/HCV-coinfected patients.

	No	IgM anti-HEV Ab.	Kappa	*p-value*	IgG anti-HEV Ab.	Kappa	*p-value*
Healthy controls	30	0/6 (0%)	0.001	**0.006**	2/5 (40%)	0.526	**0.001**
HIV-monoinfected patients	36	3/11 (27.3%)	0.342	**<0.001**	4/7(57.1%)	0.682	**<0.001**
HIV/HCV-coinfected patients	198	10/42 (23.8%)	0.330	**<0.001**	38/59 (64.4%)	0.718	**<0.001**
All subjects	264	13/59 (22.0%)	0.305	**<0.001**	44/71 (62.0%)	0.704	**<0.001**

Statistics: Values expressed as immunoblot positive/ELISA positive (%). *P*-values were calculated by Cohen’s kappa test.

Abbreviations: HEV, hepatitis E virus; HCV, hepatitis C virus; ELISA, enzyme-linked immunosorbent assay.

### HEV infection status

The seroprevalences for antibodies against HEV and their clinical interpretation are shown in Table 3. HIV/HCV-coinfected patients showed the highest prevalence of IgG antibody against HEV,resolved hepatitis E, and exposure to HEV (19.2%, 17.2%, and 22.2% respectively), but no significant differences between groups were found. Additionally, HIV/HCV-coinfected patients and HIV-monoinfected patients had the highest prevalences of anti-HEV IgM antibody and acute hepatitis E, but no significant differences from healthy controls were found. Moreover, we did not find any sample positive for HEV-RNA indicating that no patient had the possibility of chronic hepatitis E.

**Table 3:** Frequencies of immunoglobulins against HEV and clinical outcomes in the two control groups (healthy controls and HIV-monoinfected patients) and HIV/HCV-coinfected patients.

	IgM anti-HEV Ab.	IgG anti-HEV Ab.	Acute hepatitis E	Resolved hepatitis E	Exposure to HEV
Healthy controls	0 (0%)	2 (6.7%)	0/30 (0%)	2/30 (6.7%)	2/30 (6.7%)
HIV-monoinfected patients	3 (8.3%)	4 (11.1%)	3/36 (8.3%)	3/36 (8.3%)	6/36 (16.7%)
HIV/HCV-coinfected patients	10 (5.1%)	38 (19.2%)	10/198 (5.1%)	34/198 (17.2%)	44/198 (22.2%)
*p-value* (HC vs. HIV)	0.505	0.845	0.514	0.760	0.389
*p-value* (HC vs. HIV/HCV)	0.710	0.127	0.721	0.230	0.082
*p-value* (HIV vs. HIV/HCV)	0.692	0.295	0.692	0.277	0.560

Statistics: Values expressed as number of cases (%). *P*-values were calculated by chi-squared test or Fisher’s exact test as required. Abbreviations: HEV, hepatitis E virus; HC, healthy controls; HCV, hepatitis C virus; HIV-1, human immunodeficiency virus type 1.

### Influence of immunological status in HEV screening

**Figure 1:**
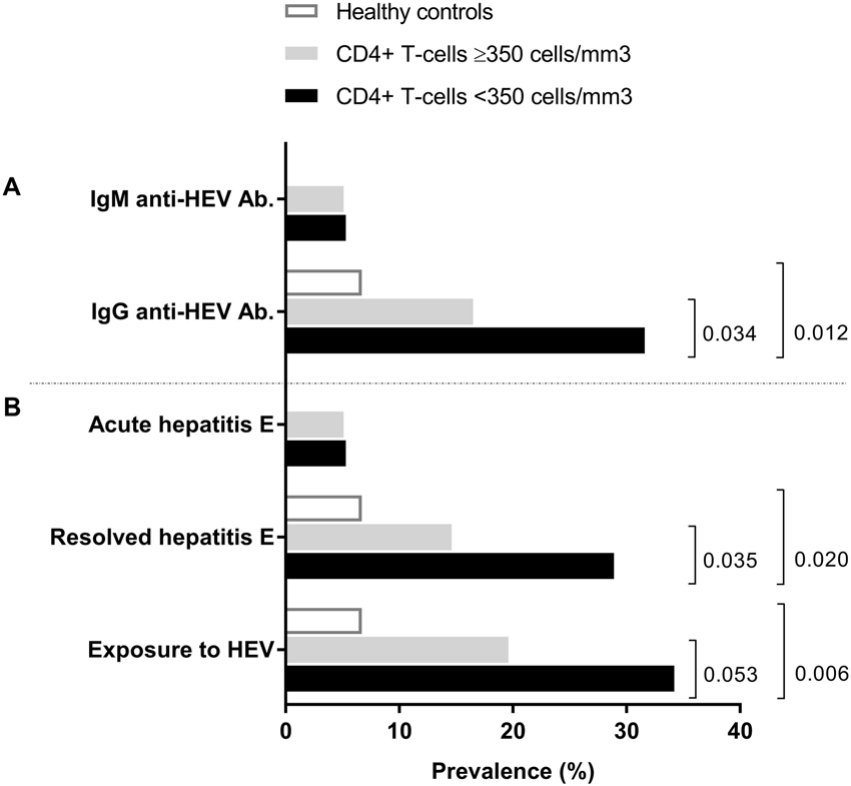
Summary of (**A**) immunoblot assay for IgM and IgG and (**B**) clinical outcomes against hepatitis E virus in HIV/HCV-coinfected patients according to CD4+ T-cell status. Statistics: Values expressed as number of cases (%). *P*-values were calculated by chi-squared test or Fisher’s exact test as required.

We found that HIV/HCV-coinfected patients with CD4 T-cells <350 cells/mm^3^ had a higher prevalence of anti-HEV IgG antibodies, resolved hepatitis E, and exposure to HEV than healthy controls (*p* = 0.012, *p* = 0.020, and *p* = 0.006; respectively) and HIV/HCV-coinfected patients with CD4 T-cells ≥350 cells/mm^3^ (*p* = 0.034, *p* = 0.035, and *p* = 0.053; respectively) (Fig. 1; full description in Supplementary Table 1). Finally, significant differences in other analyzed epidemiological and clinical characteristics were not found (*data not shown*).

## Discussion

In this work the main findings were: a) the concordance between ELISA and immunoblot assays was weak for anti-HEV IgM antibody and moderate-good for anti-HEV IgG antibody detection, making the confirmation test (immunoblot) necessary for HEV antibody screening in order to have comparable data between cohorts; b) HIV/HCV-coinfected patients had a high prevalence (>15%)of anti-HEV IgG antibodies, resolved hepatitis E, and exposure to HEV; and (c) HIV/HCV-coinfected patients with CD4+ T-cells <350 cells/mm^3^ showed a higher prevalence (>25%) for anti-HEV IgG antibodies, resolved hepatitis E, and exposure to HEV than patients with CD4+ T-cells >350 cells/mm^3^ and healthy controls. However, we did not find any relationship between HEV seroprevalence and the markers of liver disease, and no patient with a possible chronic HEV infection was found either.

There is limited data on HEV infection in HIV/HCV-coinfected patients, despite the fact that numerous studies have been published on HIV-infected patients^5^. In Spain there are several published works^7–13^ limited to a geographical area and presenting limited data in HIV/HCV-coinfected patients. This fact may lead to variability in HEV prevalence, as has already been demonstrated^16^. According to the World Health Organization and the European Centre for Disease Prevention and Control, HEV infection information for focused action is needed, particularly for those subjects considered as risk groups, such as patients with preexisting liver disease and immunocompromised status^17,18^. In this sense, our study contributes information on the HEV prevalence in a population of HIV/HCV-coinfected patients, of which 50% had cirrhosis and around 25% LSM >25 kPa (patients with high risk of clinical progression).

In our study we found disagreements between two serological techniques used for the determination of anti-HEV IgM and IgG antibodies (ELISA vs. immunoblot), reinforcing previous reports on the difficulties in HEV diagnosis^4^. These discrepancies were higher in anti-HEV IgM antibodies than anti-HEV IgG antibodies. The cause of this discordance is poorly understood, but the genetic variability of HEV and the differences in the antigens used for HEV diagnosis could explain some of these differences, as well as the lack of a reliable gold standard that would provide confidence about a true “positive” or “negative” result^4^. In other studies, cross-reactivity in anti-HEV serology tests has been described when there are infections with Epstein–Barr virus (EBV) and Cytomegalovirus (CMV)^19,20^, possibly due to reactive anti-HEV IgM antibodies generated by polyclonal activation of B-cells during acute human herpesviruses infections^19,20^. Moreover, the ELISA uses a selection of recombinant antigens with a determined conformation, which are not usually optimized, so these kits have high sensitivity but less specificity, resulting in an increase in the false positive rate^4^. However, this problem may be avoided by an immunoblot assay for the capture of IgM specific for HEV, since this assay tends to be more specific and thus can provide confirmation for the true positives^4^.

The serological diagnosis of HEV infection is not standardized, and commercially available assays show variability in specificity and sensitivity^4^. Thus, the HEV seroprevalence in the same cohort could vary when different assays are used, affecting the interpretation of the data. This fact makes it difficult to compare data from different studies based on the results of different assays^5^. In our study, we found that the seroprevalence of IgM and IgG antibodies against HEV in HIV/HCV-coinfected patients was higher than previous reports in the general Spanish population^10,19,21,22^, people living with HIV^8,10,12,13^, and other reports on HIV/HCV-coinfected patients^8^. And our seroprevalence data was higher even though most of the works did not include anti-HEV antibody confirmation by immunoblot, which likely would have reduced their seroprevalence data further. The study of Pineda *et al*. had a prevalence of 26% in HIV-infected patients, but this data was obtained without confirmation by immunoblot^11^. Moreover, our prevalence data in HIV/HCV-coinfected patients were also higher than previous reports of European people living with HIV^5^.

Levels of CD4+ T-cells in peripheral blood seem to be the most widely associated risk factor for HEV infection in HIV-infected patients^6,23,24^, although there are also articles that this association does not find^25^. The CD4+ T-cell counts reflect the level of immune suppression; and when CD4+ T-cells fall below 350 cells/μL, the risk of death, AIDS, and/or non-AIDS-defining conditions increases^26–29^. In our study, HIV/HCV-coinfected patients with CD4+ T-cells <350 cells/mm^3^ had a higher prevalence of anti-HEV IgG antibodies, resolved hepatitis E, and exposure to HEV than healthy controls and HIV/HCV-coinfected patients with CD4+ T-cells ≥350 cells/mm^3^. There is no clear explanation for this finding. One would think that subjects with CD4+ T-cells <350 cells/mm^3^ could have a worse immune response with lower titers of anti-HEV IgG antibodies, which could increase the false negative rate. However, we found higher seroprevalence of anti-HEV IgG antibodies in this subgroup of patients, which seems to indicate that CD4+ T-cells <350 cells/mm^3^ may predispose one to HEV infection. Moreover, this last explanation is not entirely clear since we found similar prevalence of anti-HEV IgM antibodies and acute hepatitis E in both groups of HIV/HCV-coinfected patients. Regarding this, we should not forget the possibility of false positives for anti-HEV IgM antibodies due to other concomitant infections^19,20^, as discussed above.

The detection of HEV-RNA is always recommended, but we did not find any sample with HEV-RNA in patients with anti-HEV IgM or IgG antibodies. Therefore, we could not confirm the HEV replication in any patient with acute hepatitis E. We also did not find any patient with a possible chronic hepatitis E since no study subject had both anti-HEV IgG antibodies and HEV-RNA in plasma. A possible explanation for this data is the temporary viremia of short duration during acute HEV infection with again time prior to the peak of antibodies against HEV^4^. In fact, most of the epidemiological studies published have reported very low numbers of positive results for HEV-RNA and chronic hepatitis E in healthy donors^5,25^ and HIV-infected patients^8,9,11,12,25^. Scotto *et al*. found a slightly higher frequency of chronic hepatitis HEV was present in subjects co-infected with HCV^25^. Despite this, HEV-RNA testing might offer an advantage in immunosuppressed patients with a worse antibody response to HEV^4^, although our HIV/HCV-coinfected patients did not have severe immunosuppression (CD4+ counts <200 cells/mm^3^).

Finally, we did not find any significant association between HEV seroprevalence and biomarkers of liver disease (LSM, liver stiffness, stages of liver fibrosis, and abnormal plasma liver enzymes), as previously suggested^13,20^.This could represent an added difficulty for the use of these markers for HIV/HCV-coinfected patients with HEV infection.

### Limitations of study

Firstly, this is a cross-sectional study with a limited number of patients in some of the study groups, which could result in a lack of uniformity and limit the possibility of finding significance. Secondly, we may have introduced a selection bias since the selection of patients from GESIDA 3603b was done with set criteria for starting HCV treatment according to guidelines in 2012–2014(e.g., CD4^+^ cell counts >200 cells/mm^3^, controlled HIV replication, and proper treatment adherence). Thirdly, this study was performed in HIV/HCV-coinfected patients and it would be also interesting to analyze a group of HCV-monoinfected patients as a control group to compare HEV prevalences, but we did not have access to a cohort of HCV-monoinfected patients. Besides, it lacked patients with decompensated cirrhosis to compare the HEV prevalence with other fibrosis stages.

### Conclusions

In conclusion, HIV/HCV-coinfected patients in Spain had high prevalences of IgG against HEV, resolved hepatitis E, and exposure to HEV as confirmed by immunoblot; in particular, patients with CD4+ T-cells <350 cells/mm^3^. Our data suggest the need to increase HEV surveillance to know the real magnitude of HEV infection and its clinical implications in HIV/HCV-coinfected patients.

## Patients and Methods

### Patients and Study design

We performed a cross-sectional study on 198 HIV/HCV-coinfected patients, from whom samples were collected from February 2012 to February 2014 from the cohort of “Grupo de Estudio del SIDA” (GESIDA 3603b study), and two control groups (30 healthy controls and 36 HIV-monoinfected patients). HIV/HCV-coinfected patients were candidates to start HCV therapy with peg-IFN-α/ribavirin or peg-IFN-α/ribavirin/direct-acting antivirals (DAAs) from 14 different institutions in Spain (see Appendix). A detailed description of the GESIDA 3603b study was previously reported^15^.

The study was conducted according to the Declaration of Helsinki, and patients gave their written informed consent to participate. The study cohort received the approval of the ethics committees of the participating centers. The Institutional Review Board and the Committee for Ethical Research (CEI) of the *Instituto de Salud Carlos III* (ISCIII) also approved the study.

### Clinical data

The information of each patient was collected from medical records, as we have previously described^15^. All information was recorded using an online form in a shared database, which included all demographic, clinical, virological and laboratory data.

A liver stiffness measurement (LSM) was performed by transient elastography (FibroScan®, Echosens, Paris, France), as we have previously described^15^. Patients were stratified according to the following LSM cutoffs: <7.1 kPa (F0-F1), 7.1–9.4 kPa (F2; significant fibrosis), 9.5-12.4 kPa (F3; advanced fibrosis), 12.5 to 25 kPa (non-risk of bleeding varices), 25 to 40 kPa (risk of bleeding varices), and >40 kPa (risk of hepatic decompensation).

### HEV antibodies assays

Plasma samples were collected at the Spanish HIV HGM BioBank and stored at −80 °C until use. Samples were tested for HEV antibodies (IgM and IgG) by enzyme-linked immunosorbent assay (ELISA) using the Abbia HEV IgM and Abbia HEV IgG kits (AB Diagnostic Systems GmbH, Germany), following the manufacturer’s instructions, on an ETI-Max 3000 instrument (DiaSorin, Saluggia, Italy). All samples positive in the ELISA for IgM and IgG were subsequently confirmed using recomLine HEV IgG/IgM kit (MIKROGEN DIAGNOSTIK, Germany) using 20 µl per sample and following manufacturer’s instructions in an Auto-LiPA 48 device (INNOGENETICS®, Siemens Healthcare Diagnostic S.L.). We include a positive control (antibodies and RNA-HEV positives from an HEV-infected patient) in order to confirm the correct performance of the techniques and HEV detection.

### Viral RNA extraction

All samples with anti-HEV IgM/IgG antibodies were tested for HEV-RNA, which was extracted from 200 ml of plasma using a commercial DSP Virus/Pathogen mini kit (Qiagen, Hilden, Germany) in the QIAsymphony instrument(Qiagen, Hilden, Germany) and stored until use at −80 °C.

### RT-PCR and Nested for HEV RNA detection

All samples from patients with anti-HEV IgM/IgG antibodies were tested for HEV genome detection using a single-step retro-transcription and primary amplification with the RT-PCR One-Step kit (Qiagen, Hilden, Germany) followed by nested PCR. A total of 5 µl of viral RNA extract was added to the RT-PCR mixture, which contained the following: 10 µl of 5X QIAGEN One-Step RT-PCR Buffer, 2 µl of dNTPs mix 10 mM, 0.25 µl of Rnase inhibitor 0.2 U/µl, 3 µl forward primer HEV1F 5′-CCAYCAGTTYMTHAAGGCTC-3′ (10 µM) and reverse primer HEV1R 5′-TRCCAVCGCTGRACRTC-3′ (10 µM), 2 µl of QIAGEN One-Step RT-PCR Enzyme mix, and nuclease-free water to a final volume of 45 μl. All reagents except primers (Sigma), and RNase inhibitor (ROCHE) were supplied with the kit. Amplification was programmed as follows: 30 min at 50 °C; 15 min at 95 °C; 40 repetitive cycles of 35 sec at 94 °C, 45 sec at 52 °C and 1 min at 72 °C; a final extension during 10 min at 72 °C.

Nested PCR was performed using 2 μl of the primary amplification product added to a mix containing 5 μl of 60% sucrose-0.08% cresol red, 5 μl of 10X PCR buffer 2w/15 mM MgCl2, 2 μl of 25 mM MgCl2, 1 μl of dNTPs 10 mM, 2 μl of each primer at 10 μM (ORF1FN and ORFIRN, previously published^19^), 0.75 μl of expand HiFi enzyme, and RNase free water up to 48 μl. All reagents except primers, 60% sucrose-0.08% cresol red and dNTPs were supplied with the Roche Expand High Fidelity System kit (Roche). The thermal conditions were 4 min at 94 °C; 30 repetitive cycles of 35 sec at 94 °C, 45 sec at 48 °C, 45 sec at 72 °C with a final extension of 5 min at 72 °C. Negative and positive controls were included in all amplification procedures. PCR products were visualized on a 2% agarose gel containing 0.1 μl/ml of 10,000X SYBR safe (Invitrogen). Positive samples showed a HEV specific band size of ~172 bp. To avoid carryover contamination, standard precautions were taken. Different biosafety cabinets were used for extraction, mixing, RT-PCR and Nested PCR and pipetting was performed with aerosol-resistant tips. Moreover, amplicons were detected in a different room.

### Clinical outcomes

The clinical interpretation of HEV screening was as follows: i) acute hepatitis E: a patient had acute hepatitis E when positive for anti-HEV IgM antibodies or both IgM and IgG, and/or HEV-RNA was detected; ii) resolved hepatitis E: a patient had resolved hepatitis E when only anti-HEV IgG antibodies were detected; and iii) exposure to HEV: a patient was exposed to HEV when acute or resolved hepatitis E was detected. This interpretation was made based on data confirmed by immunoblot since all patients positive for IgM came out negative for viral detection.

### Statistical analysis

Analyses were performed using IBM SPSS Statistics for Windows, Version 21.0 (IBM Corp, Armonk, NY, USA) and OpenEpi (http://www.openepi.com/Menu/OE_Menu.htm)^30^. All *p*-values were two-tailed and statistical significance was defined as *p* < 0.05.

For the descriptive study, values were expressed as absolute number (percentage) and median (25th; 75th percentile). Categorical data and proportions were analyzed using the chi-squared test or Fisher’s exact test. Mann-Whitney test was used to compare data among independent groups. The inter-rater agreement or concordance between serological techniques was measured by Cohen’s kappa coefficient.

## Supplementary information

Supplementary Table
