# Supplementary material for: Prevalence of hepatitis E infection in HIV/HCV-coinfected patients in Spain (2012–2014)
Source: Sci Rep. 2019 Feb 4;9:1143. doi: 10.1038/s41598-018-37328-6 (PMC6361883; doi:10.1038/s41598-018-37328-6)
Supplement: Supplementary file 1 — Supplementary Table [file 41598_2018_37328_MOESM1_ESM.pdf]

TITLE PAGE

Type of manuscript: Original Article

Title: Prevalence of hepatitis E infection in HIV/HCV-coinfected patients in Spain (2012-2014)

Authors: Sonia VÁZQUEZ-MORÓN[1*], Juan BERENGUER [2,3], Juan GONZÁLEZ-GARCÍA [4], Mª Ángeles JIMÉNEZ-SOUSA [1], Isabel CANOREA[1], Josep M GUARDIOLA[5], Manuel CRESPO [6], Carmen QUEREDA[7], José SANZ[8], Ana CARRERO[2,3], Victor HONTAÑÓN [4], Ana AVELLÓN[1], Salvador RESINO [1*]

Authors' affiliations:

(1) Laboratorio de Referencia e Investigación en Hepatitis Víricas, Centro Nacional de Microbiología, Instituto de Salud Carlos III, Majadahonda, Madrid, Spain.

(2) Unidad de Enfermedades Infecciosas/VIH; Hospital General Universitario "Gregorio Marañón", Madrid, Spain.

(3) Instituto de Investigación Sanitaria del Gregorio Marañón, Madrid, Spain.

(4) Unidad de VIH; Servicio de Medicina Interna, Hospital Universitario "La Paz"/IdiPAZ, Madrid, Spain.

(5) Hospital Santa Creu i Sant Pau, Barcelona, Spain

(6) Complexo Hospitalario Universitario; Fundación IIS Galicia Sur, Vigo; Pontevedra, Spain

(7) Hospital Universitario Ramón y Cajal. Madrid, Spain

(8) Hospital Universitario Príncipe de Asturias. Alcalá de Henares, Madrid, Spain

Corresponding author:

*Salvador Resino; Centro Nacional de Microbiología, Instituto de Salud Carlos III (Campus Majadahonda); Carretera Majadahonda- Pozuelo, Km 2.2; 28220 Majadahonda (Madrid); Phone: +34918223266. E-mail: sresino@isciii.es

*Sonia Vázquez Morón; Centro Nacional de Microbiología, Instituto de Salud Carlos III (Campus Majadahonda); Carretera Majadahonda- Pozuelo, Km 2.2; 28220 Majadahonda (Madrid); Phone: +34918223861. E-mail: svazquez@isciii.es

Supplemental Table 1.Summary of immunoblot assay for IgM and IgG and clinical outcomes against hepatitis E virusin HIV/HCV-coinfected patients according toCD4+ T-cell status.

	Healthy controls	CD4+ T-cells ≥350 cells/mm^3	CD4+ T-cells <350 cells/mm^3	*p*-value (a)	*p*-value (b)	*p*-value (c)
**No.**	30	158	38			
**IgM against HEV**	0 (0%)	8 (5.1%)	2 (5.3%)	0.483	0.617	0.999
**IgG against HEV**	2 (6.7%)	26 (16.5%)	12 (31.6%)	0.265	**0.012**	**0.034**
**Acute hepatitis E**	0 (0%)	8 (5.1%)	2 (5.3%)	0.483	0.617	0.999
**Resolved hepatitis E**	2 (6.7%)	23 (14.6%)	11(28.9%)	0.389	**0.020**	**0.035**
**Exposure to HEV**	2 (6.7%)	31 (19.6%)	13 (34.2%)	0.087	**0.006**	0.053

Statistics: Values expressed as number of cases (%). *P*-values were calculated by chi-squared test or Fisher's exact test as required. P-values: (a), healthycontrols vs. HIV/HCV-coinfected with CD4+ T-cells≥350 cells/mm3; (b), healthy controls vs. HIV/HCV-coinfected with CD4+ T-cells<350 cells/mm3; (c), HIV/HCV-coinfected with CD4+ T-cells<350 cells/mm3 vs. HIV/HCV-coinfected with CD4+ T-cells≥350 cells/mm3.
Abbreviations: HEV, Hepatitis E virus, HCV,Hepatitis C virus; HIV-1, Human immunodeficiency virus type 1.
